# Dexamethasone and tocilizumab treatment considerably reduces the value of C-reactive protein and procalcitonin to detect secondary bacterial infections in COVID-19 patients

**DOI:** 10.1186/s13054-021-03717-z

**Published:** 2021-08-05

**Authors:** Emma J. Kooistra, Miranda van Berkel, Noortje F. van Kempen, Celine R. M. van Latum, Niklas Bruse, Tim Frenzel, Maarten J. W. van den Berg, Jeroen A. Schouten, Matthijs Kox, Peter Pickkers

**Affiliations:** 1 Department of Intensive Care Medicine, Radboud University Medical Center, Postbus 9101, 6500 HB Nijmegen, The Netherlands; 2 Radboud Center for Infectious Diseases, Radboud University Medical Center, 6500 HB Nijmegen, The Netherlands; 3 Department of Laboratory Medicine, Radboud University Medical Center, 6500 HB Nijmegen, The Netherlands

**Keywords:** COVID-19, Procalcitonin, c-reactive protein, Prediction, Dexamethasone, Tocilizumab

## Abstract

**Background:**

Procalcitonin (PCT) and C-reactive protein (CRP) were previously shown to have value for the detection of secondary infections in critically ill COVID-19 patients. However, since the introduction of immunomodulatory therapy, the value of these biomarkers is unclear. We investigated PCT and CRP kinetics in critically ill COVID-19 patients treated with dexamethasone with or without tocilizumab, and assessed the value of these biomarkers to detect secondary bacterial infections.

**Methods:**

In this prospective study, 190 critically ill COVID-19 patients were divided into three treatment groups: *no dexamethasone, no tocilizumab (D−T−)*, *dexamethasone, no tocilizumab (D*+*T−)*, and *dexamethasone and tocilizumab (D*+*T*+*)*. Serial data of PCT and CRP were aligned on the last day of dexamethasone treatment, and kinetics of these biomarkers were analyzed between 6 days prior to cessation of dexamethasone and 10 days afterwards. Furthermore, the D+T− and D+T+ groups were subdivided into secondary infection and no-secondary infection groups to analyze differences in PCT and CRP kinetics and calculate detection accuracy of these biomarkers for the occurrence of a secondary infection.

**Results:**

Following cessation of dexamethasone, there was a rebound in PCT and CRP levels, most pronounced in the D+T− group. Upon occurrence of a secondary infection, no significant increase in PCT and CRP levels was observed in the D+T− group (*p* = 0.052 and *p* = 0.08, respectively). Although PCT levels increased significantly in patients of the D+T+ group who developed a secondary infection (*p* = 0.0003), this rise was only apparent from day 2 post-infection onwards. CRP levels remained suppressed in the D+T+ group. Receiver operating curve analysis of PCT and CRP levels yielded area under the curves of 0.52 and 0.55, respectively, which are both markedly lower than those found in the group of COVID-19 patients not treated with immunomodulatory drugs (0.80 and 0.76, respectively, with *p* values for differences between groups of 0.001 and 0.02, respectively).

**Conclusions:**

Cessation of dexamethasone in critically ill COVID-19 patients results in a rebound increase in PCT and CRP levels unrelated to the occurrence of secondary bacterial infections. Furthermore, immunomodulatory treatment with dexamethasone and tocilizumab *considerably reduces* the value of PCT and CRP for detection of secondary infections in COVID-19 patients.

**Supplementary Information:**

The online version contains supplementary material available at 10.1186/s13054-021-03717-z.

## Introduction

Coronavirus Disease 2019 (COVID-19) is characterized by inflammatory damage to various tissues, particularly the lung. Hence, a wide range of circulating inflammatory markers are elevated in COVID-19 patients, correlating with disease severity and outcomes [1]. This observation also holds true for procalcitonin (PCT) and C-reactive protein (CRP) [2, 3]. As has been studied repeatedly in non-COVID-19 patients, these biomarkers also have discriminatory potential for bacterial (super)infections in critically ill patients and are frequently used to assist antibiotic clinical decision making [4, 5]. In addition, once antibacterial therapy is started, repeated measurements of PCT every 48–72 h may help guide the duration of therapy [6].

We previously investigated the natural course of PCT and CRP and their value to identify secondary infections in critically ill COVID-19. We showed that COVID-19 patients have elevated concentrations at ICU admission, that gradually decline, while a later increase in these biomarkers indicate a secondary bacterial infection [5]. However, since then, pharmacological treatment of COVID-19-patients admitted to the ICU has changed considerably. The immunomodulatory drugs dexamethasone [7, 8] and the human anti-interleukin (IL)-6 receptor antibody tocilizumab [9, 10] have been shown to exert beneficial clinical effects in patients with severe COVID-19 and consequently have become part of standard care. The effects of these therapies on PCT and CRP levels in critically ill COVID-19 patients are largely unclear, but were previously assessed in non-COVID-19 patients. For instance, in patients with severe community-acquired pneumonia, corticosteroids attenuate induction of CRP, while the suppressive effect on PCT levels appears to be less pronounced [11]. In critically ill sepsis patients, treatment with corticosteroids lead to a significant decrease in CRP levels [12]. This effect of corticosteroids is independent of SOFA scores, illustrating the direct immunomodulatory effect independent of the clinical condition of the patient [13]. Interestingly, it was also reported that withdrawal of hydrocortisone treatment results in an inflammatory rebound phenomenon in septic shock patients [14]. Like corticosteroids, tocilizumab treatment also reduced CRP levels in patients with rheumatoid arthritis and in those suffering from giant cell arteritis [15]. Also in COVID-19 patients, reduction in PCT and CRP levels was observed in patients treated with tocilizumab [16]. However, differentiation between the immunomodulatory and beneficial effect on the clinical condition of the patient is difficult. Currently, the effects of dexamethasone and tocilizumab on the kinetics of PCT and CRP in COVID-19 patients are unknown. Furthermore, whether the value to detect secondary bacterial infections of these biomarkers is jeopardized by these treatments is unclear as well. In the present study, we investigated serial PCT and CRP levels in critically ill COVID-19 patients treated with dexamethasone only or in combination with tocilizumab, and compared the natural course and accuracy to detect bacterial infections to the data obtained from patients that did not receive these immunomodulatory treatments [5].

## Material and methods

### Study design and participants

**Fig. 1:**
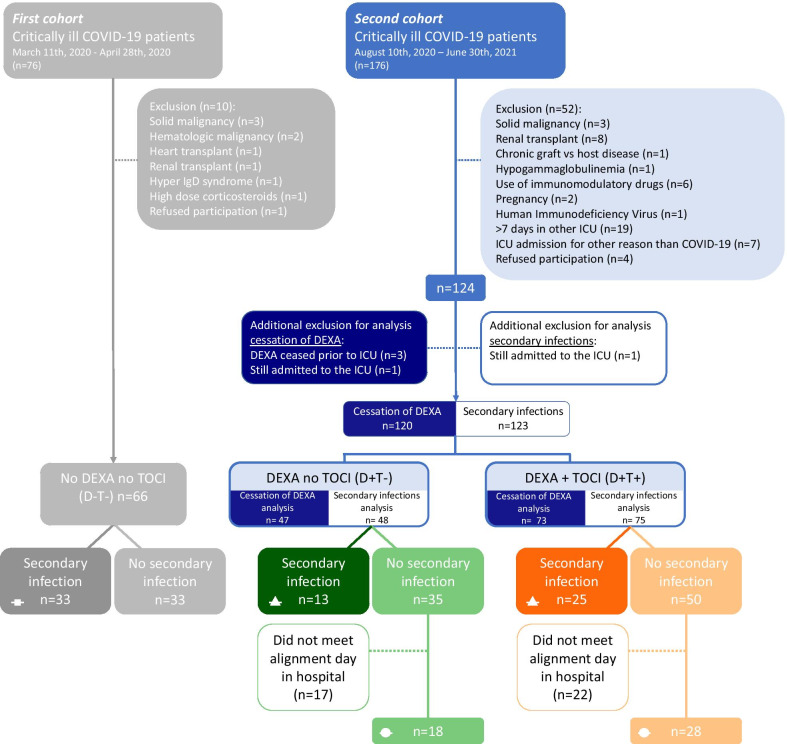
Patient flowchart. Patients who were immunocompromised based on pre-existent comorbidity or treatment and patients of the second cohort who stayed in another ICU for ≥ 7 days prior to admission to the ICU were excluded. For the analysis of PCT and CRP kinetics following cessation of dexamethasone (DEXA) treatment, patients in whom the dexamethasone treatment was already completed when admitted to the ICU, were excluded. For the analysis of PCT and CRP kinetics in patients developing a secondary infection, patients who were still admitted to the ICU on moment of data analysis (July 2021) were excluded. The remaining patients were divided into a dexamethasone-only group (D+T−) and a dexamethasone and tocilizumab (TOCI) group (D+T+), which were again subdivided into a secondary infection group and a no-secondary infection group. Following data alignment, patients in the no secondary infection groups who were discharged from the hospital on alignment day were excluded because no data was available

All patients admitted to the ICU of the Radboud university medical center (Nijmegen, The Netherlands) between March 11^th^, 2020 and April 29^th^, 2020 (‘first cohort’) and between August 10^th^, 2021 and February 5^th^, 2021 (‘second cohort’) were screened. Adult patients with COVID-19 proven by a positive SARS-CoV-2 PT-PCR test in nasopharyngeal and throat swabs were eligible for inclusion. Patients that were immunocompromised based on pre-existent comorbidity or treatment were excluded. Also, because of multiple transfers between different hospitals during the second inclusion period*,* patients who stayed in another ICU for ≥ 7 days prior to admission to the ICU of our center were not included. See Fig. 1 for an overview of the total patient selection. In the first cohort, treatment was largely supportive and patients did not receive any immunomodulating therapy. The complete second cohort received dexamethasone (DEXA) treatment as part of standard care, which was administered for a total of 10 days following hospital admission (6 mg daily, intravenously). A subgroup of the second cohort was also treated with tocilizumab (TOCI, single dose of 8 mg/kg, intravenously). First, patients were treated with tocilizumab when they were randomized in the tocilizumab subgroup of the international REMAP-CAP trial [17]. Later, when results of the REMAP-CAP trial showed beneficial effects of tocilizumab in severely ill COVID-19 patients [9], this treatment became part of standard care.

To investigate the natural course and effects of cessation of DEXA on PCT and CRP levels, patients were divided into three groups (see flowchart in Fig. 1): (1) patients from the first cohort who did not receive DEXA nor TOCI (‘D−T−’); data of this group was published previously [5], (2) patients from the second cohort who were treated with DEXA only (‘D+T−’), and (3) patients from the second cohort who were treated with both DEXA and TOCI (‘D+T+’). Patients in whom the DEXA treatment was already completed when admitted to the ICU, were excluded for this analysis. Serially measured PCT and CRP data of the latter two groups were aligned on the last day of DEXA treatment which was designated day 0. Data of the D−T− group were aligned on the median day of cessation of DEXA, relatively to ICU admission. To confirm that possibly observed effects were not based on a small group of patients with a more complicated clinical course and a longer stay in ICU, a sensitivity analysis was performed including only patients with no missing data throughout the complete study period.

In addition, to explore the predictive value of PCT and CRP to detect secondary infections in critically ill COVID-19 patients treated with DEXA with or without TOCI, patients of the second cohort were further divided into ‘secondary infection groups’ and ‘no-secondary infection groups’ (see flowchart in Fig. 1). Next to the general exclusion criteria, patients who were still admitted to the ICU on moment of data analysis (March 2021) were excluded for this analysis. A secondary bacterial infection was defined as an infectious episode confirmed by a positive culture, which was taken in case of a suspected secondary infection based on clinical signs of a new infectious episode (fever, respiratory failure, hemodynamic instability, elevated white blood cell counts). A positive culture in absence of clinical signs of a secondary infection was not scored as a secondary infection, but interpreted as colonization, as these patients were also not treated with antibiotics. The infectious episodes were determined from the electronical medical records by three ICU physicians (MvdB, TF and JS). Information about the type of infection and causative agents was retrieved from the medical records. Serially measured PCT and CRP levels of the secondary infection groups were aligned on the day the positive culture was taken, which was designated day 0. In case of multiple sequential secondary infections, only the first day of the first infectious episode was used as alignment day. Data of the no-secondary infections groups were aligned on the median day that secondary infections occurred in the affected groups following ICU admission. Following data alignment, patients in the no-secondary infection groups who were discharged from the hospital on alignment day were excluded because no data was available. To illustrate possible differences with our previously published results of the first cohort [5], data of the secondary infection group of this study (D−T−) were also included in the figures. Finally, all patients who developed a secondary infection were divided into an early infection group (secondary infection occurred ≤ 4 days following cessation of DEXA therapy) and a late infection group (secondary infection occurred > 4 days following cessation of DEXA therapy). Serially measured PCT and CRP, again aligned on day of secondary infection, were compared between the early and late infection groups to investigate whether the predictive accuracy of PCT and CRP differed between patients who developed a secondary infection during or early after DEXA treatment and patients who developed a secondary infection at a later stage in the ICU.

This study was carried out in accordance with the applicable rules concerning the review of research ethics committees and informed consent in the Netherlands. All patients or legal representatives were informed about the details of this cohort study and could decline to participate.

### Data collection

Data collection was carried out as part of a cohort study in critically ill COVID-19 patients in the ICU of the Radboud university medical center. Data of patient characteristics, medical history and clinical parameters were collected from the electronic patient files (Epic, Epic Systems Corporation, Verona, Wisconsin, USA) and recorded in the Good Clinical Practice-certified data management system Castor (Castor EDC, Amsterdam, the Netherlands). For clinical purposes, PCT and CRP were determined every 48 h. PCT was determined using the Elecsys BRAHMS procalcitonin assay (Thermo Fisher Scientific, Waltham, MA, USA) and CRP was determined using an immunoturbidimetric assay, both on a Cobas 8000 immuno-analyzer (Roche Diagnostics, Rotkreuz, Switzerland).

### Statistical analysis

Serial PCT and CRP data were aligned (see above) and binned into bins spanning two days using a script made in RStudio v3.6.2 (RStudio, PBC, Boston, USA). Differences in patient characteristics between the D−T−, D+T− and D+T+ groups were analyzed using Kruskal Wallis and chi-square tests followed by post-hoc Dunn’s multiple comparisons tests or pairwise chi-square tests, respectively. Patient characteristics of the secondary infection groups and no-secondary infection groups (within the D−T−, D+T− and D+T+ groups) were analyzed using chi-square and Mann Whitney U tests. Between-group differences over time were analyzed using linear mixed effects model analysis on log-transformed data followed by post-hoc analyses using Sidak’s multiple comparisons tests. For comparisons of PCT and CRP kinetics in patients with and without secondary infections, and to compare the early and late secondary infection groups, data were analyzed from day − 10 until day 10 relative to the day of secondary infection. For these analyses, we performed the Last Observation Carried Forward (LOCF) method for data of patients who were discharged from the hospital between day + 1 and day + 10. To illustrate sensitivity and specificity of PCT and CRP levels to predict secondary infections, receiver operating curve (ROC) analyses were performed using binned data of day − 1 and 0. Differences in area’s under the receiver operating curves (AUROCs) between patients treated with and without immunomodulatory drugs were assessed using the following strategy:

First, AUROCs and corresponding standard errors (SE) of separate groups (D−T−, D+T−, D+T+ and D+T−/+) were determined. Subsequently, z-scores for differences between AUROCs were calculated using the following formula: $z = \frac{{\left( {ROCAUC1 - ROCAUC2} \right)}}{{\sqrt {SE1^{2} + SE2^{2} } }}$.

Finally, two-tailed *p* values were determined using the following function in Excel 2016 (Microsoft Corporation): 2 × (1-NORMSDIST(Z)).

Data are displayed as number (%), median with interquartile ranges [IQR], or geometric mean with 95% confidence intervals (CI). All statistical analyses were performed in SPSS Statistics 25 (IBM SPSS Statistics, Version 25.0. Armonk, NY: IBM Corp) and Graphpad Prism 8 Software (GraphPad Software, La Jolla California USA, www.graphpad.com).

## Results

### Natural course and rebound of PCT and CRP following cessation of dexamethasone treatment

**Fig. 2:**
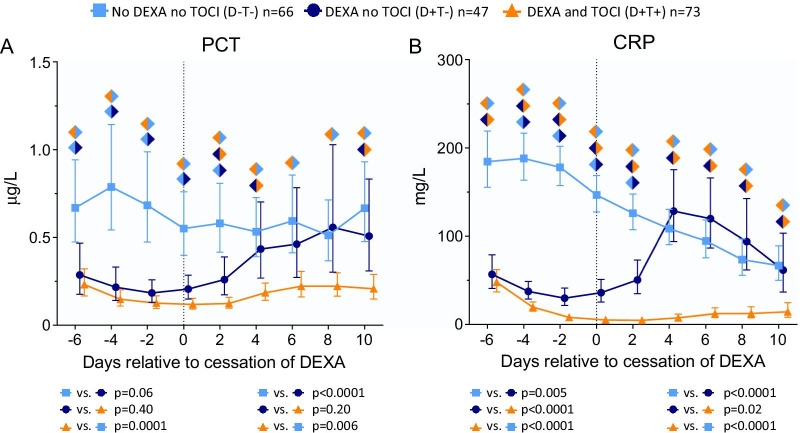
Levels of **a** procalcitonin (PCT) and **b** C-reactive protein (CRP) over time within 6 days prior to and 10 days following cessation of dexamethasone (DEXA) in the group of patients treated with neither dexamethasone nor tocilizumab (D−T− group) as well as in the D+T− and D+T+ groups. Day of cessation of dexamethasone was designated day 0 (alignment day). Data of the D−T− group were aligned on the median alignment day, which was day 9 following ICU admission. Data are presented as geometric mean with 95% confidence intervals. *p* values were calculated using mixed-models analyses (time × group interaction factor). *p* values left and right below each panel reflect between-group differences in kinetics from day − 6 until day 0 and from day 0 until day 10, respectively. Colored diamonds reflect *p* values of < 0.05 between the corresponding groups (D−T− light blue, D+T− dark blue, D+T+ orange) on the individual timepoint, calculated using Sidak’s post-hoc multiple comparisons tests. D−T−: patients treated with neither dexamethasone nor tocilizumab, D+T−: patients treated with dexamethasone but no tocilizumab, D+T+: patients treated with both drugs

We first assessed the natural course and effects of cessation of dexamethasone (DEXA) treatment on PCT and CRP levels in critically ill COVID-19 patients. This was also analyzed in DEXA-treated patients who also received tocilizumab (TOCI). Sixty-six, 47, and 73 patients were included in the no DEXA no TOCI (D−T−), DEXA no TOCI (D+T−), and DEXA and TOCI (D+T+) groups, respectively (Fig. 1). Patients in the D+T− and D+T+ groups had a significant higher body mass index (BMI) compared to those in the D−T− group (*p* = 0.02, Table 1). As a result of treatment, levels of both PCT and CRP were lower in patients in the D+T− and D+T+ groups compared to the D−T− group at ICU admission (*p* = 0.001 and *p* < 0.0001, respectively). DEXA therapy ended 9 days [7–10] following ICU admission (median [IQR]); this is designated alignment day 0 in Fig. 2. Following cessation of DEXA therapy, PCT significantly increased in the D+T− and D+T+ groups compared to the D−T− group (*p* < 0.0001 and *p* = 0.006, respectively, Fig. 2a). In the D+T− group, this rebound effect was even more pronounced for CRP levels (Fig. 2b). Compared to the continuously declining CRP levels in the D−T− group, CRP markedly increased in the D+T− group during the first four days after cessation of DEXA treatment (*p* < 0.0001). In contrast, in the D+T+ group, CRP levels did not show any rebound following day 0 and remained significantly lower compared to the D−T− and D+T− groups at all subsequent timepoints (*p* < 0.0001, Fig. 2b). To exclude the possibility that the observed rebound effects were due to the patients with a more complicated clinical course that remained in the ICU (while patients recovered were discharged), a sensitivity analysis was performed using patients with no missing data throughout the complete study period. This analysis yielded comparable rebound effects (data not shown), indicating that this effect was not due to case-mix changes.

**Table 1:** Patient characteristics and circulating levels of C-reactive protein and procalcitonin on ICU admission in the D−T−, D+T− and D+T+ groups

	No DEXA, no TOCI (D−T−, *n* = 66)	DEXA, no TOCI (D+T−, *n* = 47)	DEXA + TOCI (D+T+, *n* = 73)	*p* value
Sex, male	49 (74)	35	46	0.26
Age, years	66 [59–72]	66 [56–72]	64 [57–71]	0.77
Body mass index, kg/m^2^	27.6 [24.9–30.9]^§^	29.0 [26.3–32.4]	29.5 [26.2–34.4]	*0.02*
APACHE II	15 [12–19]	15 [13–18]	16 [14–21]	0.06
Time from first COVID-19 symptoms until ICU admission, days	11 [7–13]	9 [6–12]	10 [8–12]	0.19
*Medical history*				
Renal insufficiency	1 (2)	2	1	0.52
Metastatic neoplasm	5 (8)	1	1	0.13
Immunological insufficiency	1 (2)	2	3	0.62
COPD	6 (9)	6	7	0.80
Diabetes mellitus	15 (23)	14	17	0.65
Hypertension	33 (50)	24	38	0.97
*Biomarkers at ICU admission*				
Procalcitonin, µg/L	0.62 [0.26–1.05]*^,§^	0.24 [0.17–0.46]	0.26 [0.11–0.42]	*0.001*
C-reactive protein, mg/L	222 [133–275]*^,§^	94 [68–133]	90 [43–146]	* < 0.0001*

All statistically significant* p*-values are depicted in italics

*p* values were calculated using Kruskal Wallis or chi-square tests, followed by Dunn’s multiple comparison tests and pairwise chi-square tests, respectively. Data are presented as n (%) or median [IQR]

^*^p < 0.05 compared to D+T− group. ^§^*p* < 0.05 compared to D+T+ group

### PCT and CRP levels in patients who developed secondary infections

**Fig. 3:**
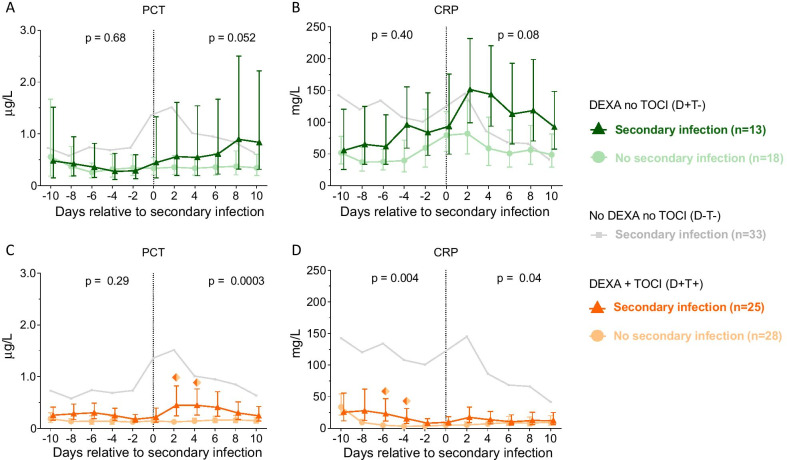
Levels of procalcitonin (PCT) and C-reactive protein (CRP) over time within 10 days prior to and 10 days following the day of secondary infection in the group of patients treated with dexamethasone (TOCI) but not with tocilizumab (TOCI, D+T− group, **a** and **b**, and in the D+T+ group (**c** and **d**). Day of secondary infection was designated day 0 (alignment day). Data of the no secondary infection groups were aligned on the median alignment day, which was day 14 following ICU admission. The light grey line indicates previously reported data of D−T− patients as a reference [5]. Data are presented as geometric mean with 95% confidence intervals. *p* values were calculated using mixed-models analyses (time × group interaction factor). *p* values in left and right parts of each panel reflect between-group differences in kinetics from day − 10 until day 0 and from day 0 until day 10, respectively. Colored diamonds reflect *p* values of < 0.05 on the individual timepoints, calculated using Sidak’s post-hoc multiple comparisons tests. D−T−: patients treated with neither dexamethasone nor tocilizumab, D+T−: patients treated with dexamethasone but no tocilizumab, D+T+: patients treated with both drugs

One hundred twenty three patients included in the second cohort, all of whom were treated with DEXA, were divided into D+T− (*n* = 48) and D+T+ (*n* = 75) groups (Fig. 1). In the D+T− group, 13 patients (27%) developed a secondary infection, whereas this was the case for 25 patients (33%) of the D+T+ group (Fig. 1). Secondary infections consisted of pulmonary tract infections and (catheter-associated) bloodstream infections with a wide variety of causative pathogens (depicted in Additional file 1: Fig. S1). No differences in patient characteristics were observed between the secondary infection and no-secondary infection groups for the D−T−, D+T− and D+T+ groups (Table 2). In patients who developed a secondary infection, a positive culture was obtained on median [IQR] day 14 [11–19] following ICU admission, which was designated day 0 for the following analyses. In contrast to the results of our previously published study in COVID-19 patients who did not receive immunomodulatory therapy [5] (depicted by the light grey line in Fig. 3a), no significant increase in PCT and CRP levels was observed in patients of the D+T− group who developed a secondary infection compared to patients who did not (*p* = 0.052 and *p* = 0.08, respectively, Fig. 3a, b). PCT levels in the D+T+ group significantly increased from day 2 onwards in patients who developed a secondary infection (Fig. 3c, p = 0.0003). In contrast, CRP induction remained completely suppressed in the D+T+ group (Fig. 3d). When comparing the early and late secondary infection groups (development of secondary infection at 1 [− 1–2] and 12 [9–16] days after cessation of DEXA, respectively), the late infection group displayed a more pronounced increase in levels of both PCT and CRP following alignment day, with significantly higher levels of PCT on days 0 and 2 (Additional file 2: Fig. S2).

**Table 2:** Patient characteristics and clinical parameters on ICU admission and on the day patients developed a secondary infection (alignment day) within the D−T−, D+T− and D+T+ groups

	No DEXA, no TOCI (D−T−)			DEXA, no TOCI (D+T−)			DEXA and TOCI (D+T+)		
	Secondary infection (*n* = 33)	No secondary infections (*n* = 33)	*p* value	Secondary infection (*n* = 13)	No secondary infections (*n* = 18)	*p* value	Secondary infection (*n* = 25)	No secondary infections (*n* = 28)	*p* value
Sex, male	26 (79)	23 (70)	0.57	9 (69)	12 (67)	1.00	19 (76)	18 (64)	0.39
Age, years	67 [60–73]	65 [56–70]	0.17	67 [59–73]	71 [60–73]	0.76	68 [59–71]	63 [56–72]	0.38
BMI, kg/m^2^	27.6 [25.4–31.1]	27.7 [24.3–30.7]	0.40	29.4 [25.5–32.5]	29.8 [27.5–32.0]	0.48	27.5 [26.0–34.4]	29.4 [26.3–35.1]	0.54
APACHE II	15 [13–19]	15 [10–19]	0.77	15 [12–22]	17 [13–21]	0.32	19 [15–22]	16 [12–22]	0.20
Time from first COVID-19 symptoms until ICU admission, days	10 [7–13]	10 [6–14]	0.96	9 [6–12]	10 [8–12]	0.45	9 [5–12]	10 [9–11]	0.40
*Medical history*									
Renal insufficiency	0 (0)	1 (3)	1.00	1 (8)	1 (6)	1.00	0 (0)	1 (4)	1.00
Metastatic neoplasm	2 (6)	3 (9)	1.00	0 (0)	1 (6)	1.00	1 (4)	0 (0)	0.47
Immunological insufficiency	0 (0)	1 (3)	1.00	0 (0)	1 (6)	1.00	1 (4)	3 (11)	0.61
COPD	3 (9)	3 (9)	1.00	1 (8)	4 (22)	0.37	3 (12)	3 (11)	1.00
Diabetes mellitus	11 (33)	4 (12)	0.08	4 (31)	8 (44)	0.48	7 (28)	7 (25)	1.00
Hypertension	17 (52)	16 (48)	1.00	8 (62)	12 (67)	1.00	13 (52)	15 (54)	1.00
*Clinical parameters on alignment day*									
Temperature, Celsius	38.9 [37.8–40.0]	38.2 [37.3–38.9]	*0.03*	38.0 [37.2–38.6]	37.6 [36.7–38.2]	0.33	37.0 [36.7–38.6]]	37.3 [36.9–37.9]	0.90
Leukocytes, × 10^9^/L	16.1 [12.9–20.2]	11.7 [9.3–13.0]	*0.001*	10.7 [8.1–16.1]	10.9 [9.9–14.5]	1.00	10.0 [7.3–14.6]	9.1 [6.1–14.6]	0.48

All statistically significant* p*-values are depicted in italics

Data of the no secondary infection groups were aligned on the median day following ICU admission that the secondary infections occurred in the affected groups. *p* values were calculated using Mann–Whitney or Chi-square tests. Data are presented as n (%) or median [IQR]

### Value of PCT and CRP for early detection of secondary infections

**Fig. 4:**
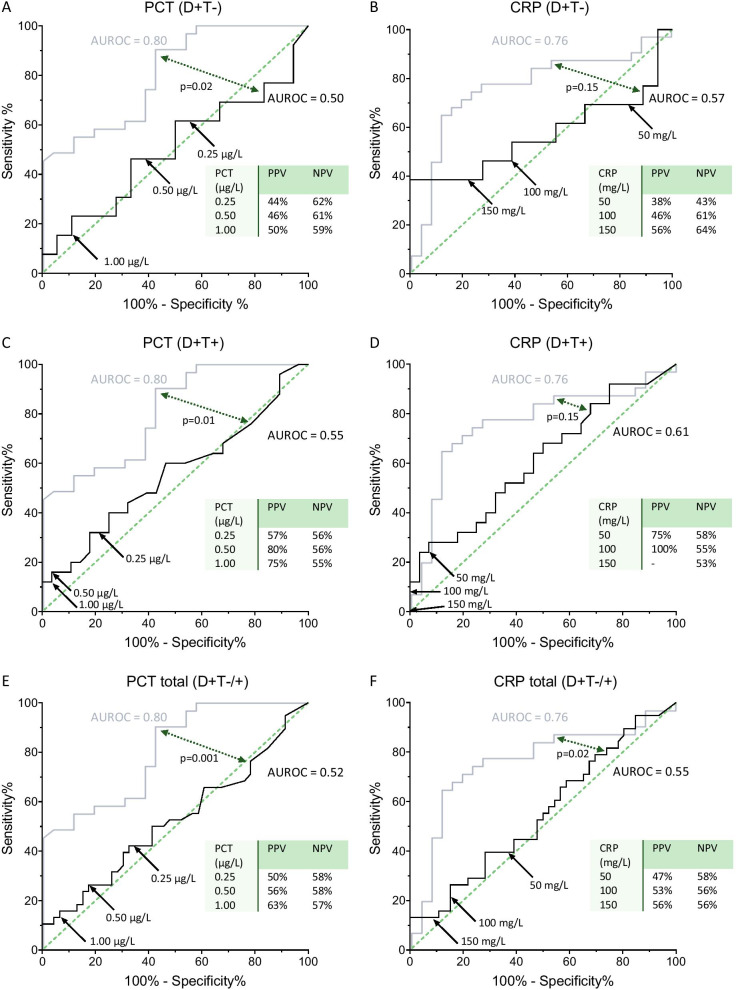
Receiver operating curves (ROC) of procalcitonin (PCT) and C-reactive protein (CRP) in the group of patients treated with dexamethasone but not with tocilizumab (D+T− group, **a** and **b**), the D+T+ group (**c** and **d**), and in all patients of the second cohort (D+T−/+ group, **e** and **f**) to illustrate sensitivity and specificity to predict the occurrence of a secondary infection in critically ill COVID-19 patients. Binned data of PCT and CRP of days − 1 and 0 were used for these analyses. The grey lines illustrate the previously published ROCs of D−T− patients [5]. *p* values reflect differences between the two areas under the receiver operating curves (AUROCs). Positive predictive value (PPV) and negative predictive value (NPV) are provided for the concentrations of PCT and CRP indicated by the arrows

The value of PCT and CRP to detect secondary infections in critically ill COVID-19 patients treated with DEXA was investigated for both D+T− and D+T+ groups as well as for the total second cohort (D+T−/+). In the D+T− group, receiver operating curve (ROC) analysis of PCT and CRP levels on the day of secondary infection yielded area under the receiver operating curves (AUROC) of 0.50 and 0.57, respectively (Fig. 4a, b). These AUROCs are markedly lower than those reported in our previous study in COVID-19 patients not treated with DEXA (0.80 and 0.76, *p* = 0.02 and *p* = 0.15, respectively) [5]. Data of the D+T+ group showed comparable results, with AUROCs of 0.55 and 0.61 for PCT and CRP, respectively (*p* = 0.01 and *p* = 0.15, respectively, Fig. 4c, d). In the D+T−/+, the AUROC of PCT was 0.52 (*p* = 0.001 compared to AUROC of D−T−, Fig. 4e), whereas the AUROC of CRP was 0.55 (*p* = 0.02 compared to AUROC of D−T−, Fig. 4f). In accordance, positive predictive value (PPV) and negative predictive value (NPV) were lower compared to patients from the first cohort who did not receive immunomodulating therapy [5]. In the D+T+ group, PPV could not be calculated because no patients displayed CRP levels > 150 mg/L.

## Discussion

In the present study, we investigated the effects of dexamethasone and tocilizumab treatment on PCT and CRP kinetics, as well as the values of these inflammatory biomarkers for early detection of secondary infections in critically ill COVID-19 patients. We demonstrate that PCT and CRP levels are suppressed by dexamethasone treatment and that, after completion of the dexamethasone course, a clear inflammatory rebound effect was observed for both biomarkers, but particularly for CRP. In patients treated with both dexamethasone and tocilizumab, PCT also increased following cessation of dexamethasone, albeit less pronounced than in patients treated with dexamethasone only. Combined treatment with dexamethasone and tocilizumab resulted in suppressed CRP levels, an effect which persisted for the total observation period. Finally, the value of both biomarkers for the early detection of secondary infections was *considerably reduced* following immunotherapy.

In accordance with our results, a meta-analysis of biomarker levels before and after administration of tocilizumab in COVID-19 patients showed a reduction of CRP and a -non statistically significant-reduction of PCT [16]. Also, dexamethasone therapy resulted in markedly reduced CRP levels in COVID-19 patients [18]. However, except for one explorative study describing reduced inflammatory markers in COVID-19 patients treated with immunomodulatory drugs as a secondary endpoint [19], no studies have related overall suppression of PCT and CRP to their ability to detect secondary bacterial infections in COVID-19 patients. Furthermore, while so-called ‘rebound effects’ on inflammatory biomarkers after cessation of corticosteroid therapy have been observed in other conditions [14], they have not been studied in COVID-19 patients yet. The relatively swift CRP rebound after cessation of dexamethasone therapy is in line with its biological half-life of 36–54 h [20]. A sensitivity analysis indicating that the rebound effect is not caused by differences in case mix strengthens this finding. The observed rebound-effect in COVID-19 patients is of clinical relevance, as it reflects a potential false positive signal for the development of a secondary infection. In addition, despite the apparent return of PCT and CRP levels to elevated levels after cessation of dexamethasone therapy, a further increase caused by secondary infections remained limited over the entire observation period and consequently we observed that these biomarkers lost their diagnostic ability to detect secondary infections, representing a false negative signal. This may be explained by other, more prolonged anti-inflammatory effects of dexamethasone [21]. The long half-life of tocilizumab (approximately 10 days) explains the much more prolonged suppression of both PCT and CRP levels observed in our study and the complete absence of a CRP rebound-effect.

Our study clearly demonstrates that the values of PCT and CRP to detect secondary infections in COVID-19, which we previously showed to be helpful in patients who did not receive immunomodulatory treatment [5], is *considerably reduced* by use of dexamethasone, whether or not in combination with tocilizumab. The potent and long-lasting anti-inflammatory effects of dexamethasone and tocilizumab appear to directly attenuate PCT and CRP to such extent that they are no longer sufficiently induced in response to bacterial infection. These findings take us back to the question often asked in daily ICU practice: how can we reliably recognize ICU-acquired infections and hence decide on appropriate antimicrobial treatment? The increased use of immunomodulatory agents in the ICU, during COVID-19 times but also in our non-COVID-19 population prevents the use of an important tool for antimicrobial stewardship in the ICU. In an era of increasing antimicrobial resistance due to consistent overuse of antibiotics in ICU, we are now thrown back to ‘basic clinical reasoning’ when deciding to start antimicrobial treatment or not. Our findings indicate that decisions based on the levels of these biomarkers may be false positive (rebound effect) as well as false negative (especially within 4 days following cessation of immunomodulatory treatment). Interestingly, when comparing patients who developed a secondary infection early after cessation of dexamethasone (≤ 4 days) to patients who developed such an infection later on (> 4 days), a more pronounced increase in PCT and CRP was apparent in the late infection group. These findings may indicate that both biomarkers regain some value to detect secondary infections at a later stage after cessation of dexamethasone therapy in critically ill COVID-19 patients. Further research is needed to confirm this hypothesis. Also in decision making on stopping antibiotic therapy [6], immunomodulatory drugs likely affect the predictive characteristics of PCT and CRP, but this still needs to be confirmed. Nevertheless, since a delayed peak in CRP was observed within 2–4 days following the day of secondary infection in patients treated with only dexamethasone (not tocilizumab), the absence of an increase in CRP during the first 2–4 days could possibly be used in the decision to cease antibiotic therapy.

This study has several limitations. First, the observational design with two different periods of inclusion may lead to possible time-related bias when comparing these groups. It cannot be excluded that the differences between both groups can be partly explained by the fact that knowledge about COVID-19 improved over time and medical staff have become more experienced in caring for COVID-19 patients. Second, steroid treatment in the ward likely prevented most patients from deteriorating, implying that selection bias for those patients that did not respond and were transmitted to the ICU might be present. This might have resulted in a different population in the ICU and different outcomes and complications, although no major differences in disease severity, clinical parameters and patient characteristics between groups on ICU admission were observed. Third, the relatively small number of patients in our cohort resulted in rather limited sample sizes after further division into different subgroups (e.g., secondary infection vs. no-secondary infection). Nevertheless, this is, to our knowledge, the first study to assess the predictive value of PCT and CRP for detection of secondary infection after the introduction of dexamethasone and tocilizumab treatment in critically ill COVID-19 patients. Finally, in the present study we focused on well-established biomarkers of bacterial infection. Therefore, it remains to be determined to what extent the immunomodulatory treatment would influence the predictive value of other markers or for other types of infections, such as beta-d-glucan and galactomannan for invasive candidiasis and CAPA.

## Conclusions

Our study shows that in critically ill COVID-19 patients, the inflammatory biomarkers CRP and PCT show a rebound increase upon cessation of dexamethasone treatment, potentially leading to false-positive findings. Furthermore, the use of immunomodulatory treatments in critically ill COVID-19 patients *considerably reduces* the value of PCT and CRP for detection of secondary infections, reflecting a false-negative finding. As a result, clinicians should not rely on these biomarkers, but assess basic clinical infection signs and cultures to detect secondary infections in COVID-19 patients that received these immunomodulating treatments.

## Supplementary Information

**Additional file 1. Fig. S1**: Pie charts illustrating the sites of secondary bacterial infections and the causative pathogens in the second cohort (patients treated with dexamethasone with or without tocilizumab, D+T-/+ group).

**Additional file 2. Fig. S2**: Levels of A) procalcitonin (PCT) and B) C-reactive protein (CRP) over time within 10 days prior to and 10 days following the day of secondary infection in all patients of the second cohort treated with dexamethasone with or without tocilizumab (D+T-/+ group) who developed a secondary infection early (≤4 days) and late (> 4 days) following cessation of dexamethasone. Day of secondary infection was designated day 0 (alignment day). Data are presented as geometric mean with 95% confidence intervals. P-values were calculated using mixed-models analyses (time*group interaction factor). P-values in left and right parts of each panel reflect between-group differences in kinetics from day -10 until day 0 and from day 0 until day 10, respectively. Colored diamonds reflect p-values of < 0.05 on the individual timepoints, calculated using Sidak’s post-hoc multiple comparisons tests.

## Data Availability

The datasets used and/or analyzed during the current study are available from the corresponding author on reasonable request.

## Abbreviations

AUROC: Area under the receiver operating curve
CI: Confidence intervals
COVID-19: Coronavirus disease 2019
CRP: C-reactive protein
DEXA: Dexamethasone
ICU: Intensive care unit
IL: Interleukin
IQR: Interquartile ranges
NPV: Negative predictive value
PCT: Procalcitonin
PPV: Positive predictive value
ROC: Receiver operating curve
TOCI: Tocilizumab
