# Supplementary figures and images for: Dexamethasone and tocilizumab treatment considerably reduces the value of C-reactive protein and procalcitonin to detect secondary bacterial infections in COVID-19 patients
Source: Crit Care. 2021 Aug 5;25:281. doi: 10.1186/s13054-021-03717-z (PMC8340482; doi:10.1186/s13054-021-03717-z)

## INFECTION SITE

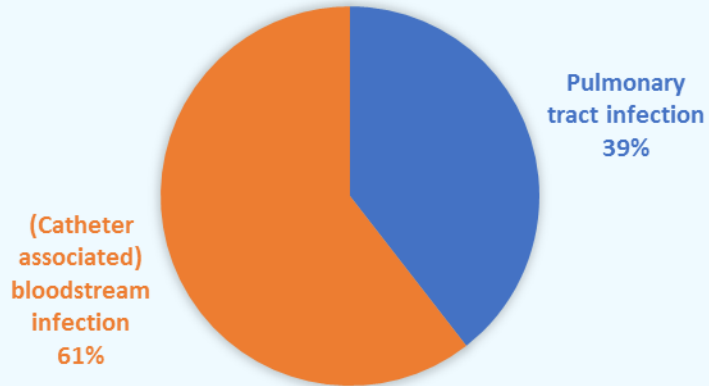

## CAUSATIVE PATHOGENS

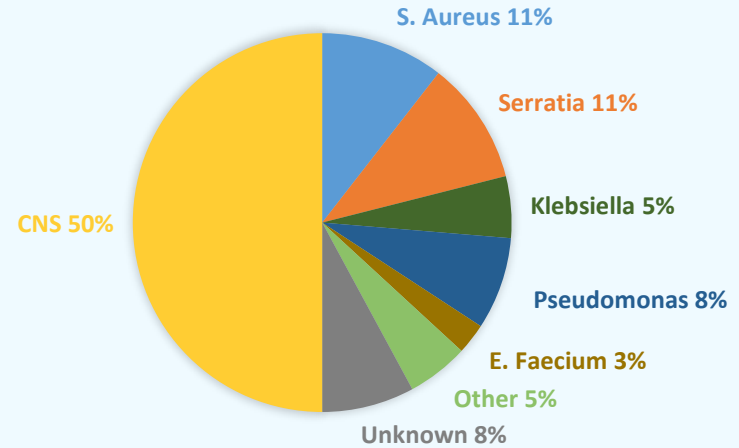

Supplement: Supplementary file 1 — Additional file 1. Fig. S1: Pie charts illustrating the sites of secondary bacterial infections and the causative pathogens in the second cohort (patients treated with dexamethasone with or without tocilizumab, D+T-/+ group). [file 13054_2021_3717_MOESM1_ESM.pdf]

■ Early secondary infection ( $\leq 4$  days)

● Late secondary infection ( $>4$  days)

PCT

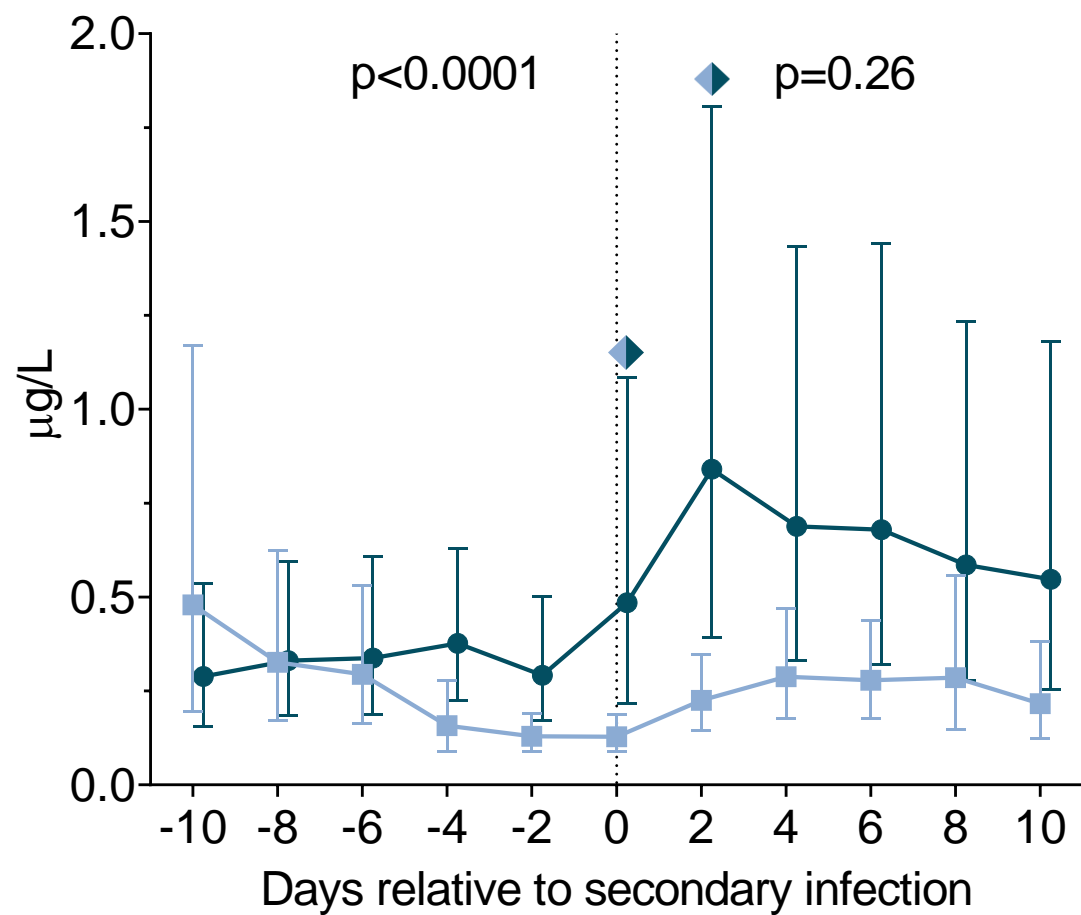

CRP

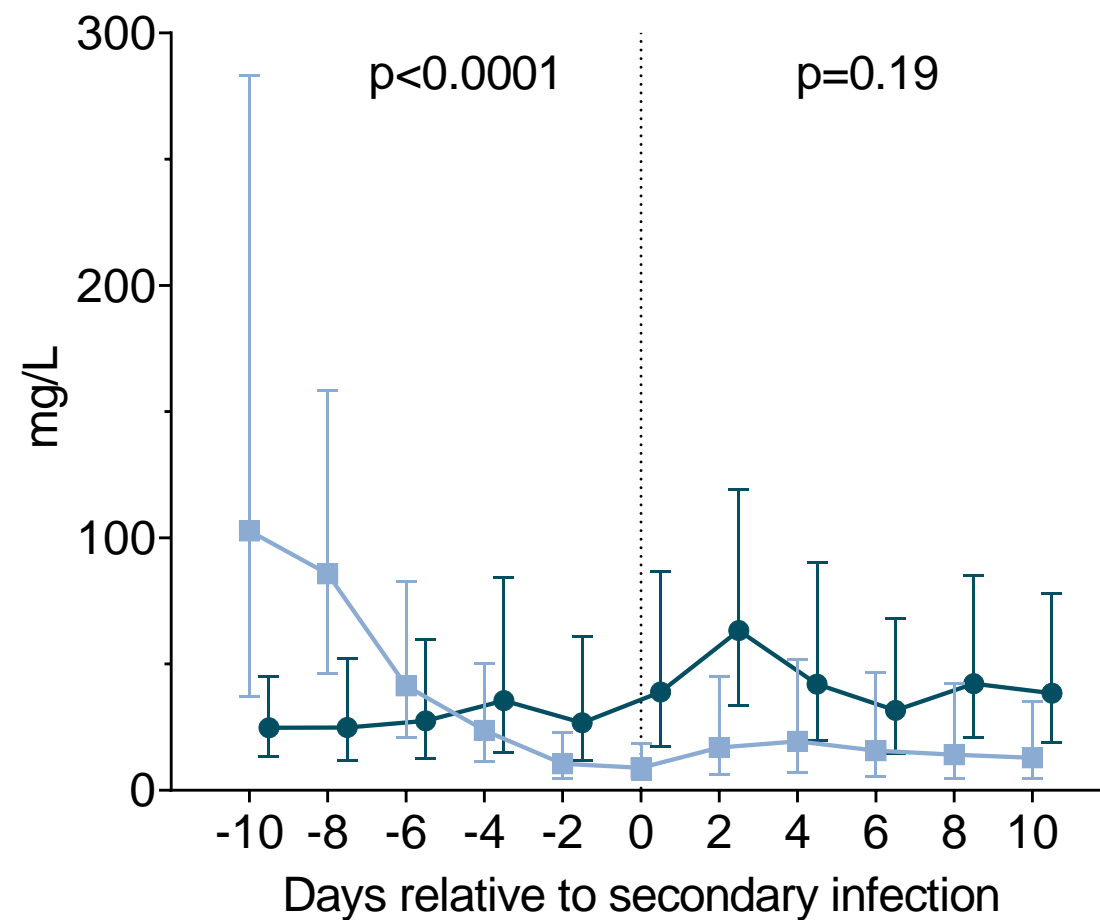

Supplement: Supplementary file 2 — Additional file 2. Fig. S2: Levels of A) procalcitonin (PCT) and B) C-reactive protein (CRP) over time within 10 days prior to and 10 days following the day of secondary infection in all patients of the second cohort treated with dexamethasone with or without tocilizumab (D+T-/+ group) who developed a secondary infection early (≤4 days) and late (> 4 days) following cessation of dexamethasone. Day of secondary infection was designated day 0 (alignment day). Data are presented as geometric mean with 95% confidence intervals. P-values were calculated using mixed-models analyses (time*group interaction factor). P-values in left and right parts of each panel reflect between-group differences in kinetics from day -10 until day 0 and from day 0 until day 10, respectively. Colored diamonds reflect p-values of < 0.05 on the individual timepoints, calculated using Sidak’s post-hoc multiple comparisons tests. [file 13054_2021_3717_MOESM2_ESM.pdf]
